# How Will Artificial Intelligence Shape the Future of Decision-Making in Congenital Heart Disease?

**DOI:** 10.3390/jcm13102996

**Published:** 2024-05-20

**Authors:** Alice Pozza, Luca Zanella, Biagio Castaldi, Giovanni Di Salvo

**Affiliations:** 1 Paediatric Cardiology Unit, Department of Women’s and Children’s Health, University of Padua, 35122 Padova, Italy; alice.pozza@aopd.veneto.it (A.P.);; 2 Heart Surgery, Department of Medical and Surgical Sciences, University of Bologna, 40138 Bologna, Italy; 3 Cardiac Surgery Unit, Department of Cardiac-Thoracic-Vascular Diseases, IRCCS Azienda Ospedaliero-Universitaria di Bologna, 40138 Bologna, Italy

**Keywords:** congenital heart disease, artificial intelligence, machine learning, tailored treatment

## Abstract

Improvements in medical technology have significantly changed the management of congenital heart disease (CHD), offering novel tools to predict outcomes and personalize follow-up care. By using sophisticated imaging modalities, computational models and machine learning algorithms, clinicians can experiment with unprecedented insights into the complex anatomy and physiology of CHD. These tools enable early identification of high-risk patients, thus allowing timely, tailored interventions and improved outcomes. Additionally, the integration of genetic testing offers valuable prognostic information, helping in risk stratification and treatment optimisation. The birth of telemedicine platforms and remote monitoring devices facilitates customised follow-up care, enhancing patient engagement and reducing healthcare disparities. Taking into consideration challenges and ethical issues, clinicians can make the most of the full potential of artificial intelligence (AI) to further refine prognostic models, personalize care and improve long-term outcomes for patients with CHD. This narrative review aims to provide a comprehensive illustration of how AI has been implemented as a new technological method for enhancing the management of CHD.

## 1. Introduction

Congenital heart disease (CHD) is the most common congenital anomaly affecting millions of individuals worldwide, occurring in nearly 1% of live births [1].

Improvements in diagnostic accuracy and surgical techniques have allowed children affected by CHD to not just survive but most of all, thrive well into adulthood. Nevertheless, the management of these patients might be challenging due to the merging of post-surgical anatomy in complex CHD and acquired diseases [2,3]. CHD presents a complex clinical landscape, requiring intricate management strategies tailored to each patient’s unique needs [4].

Traditionally, follow-up care is based on conventional protocols, providing an overview of anatomical and functional features. However, outcome prediction has been based on clinical and anatomical clusters, which may not fully represent real-life scenarios.

To overcome these limitations, there is a growing interest in further exploring heart structures by implementing the use of novel technologies in the standard diagnostic workflow and follow-up of these patients [5,6]. Recently, artificial intelligence (AI) has experienced great success in the scientific area, including healthcare [7]. Paediatric cardiology can benefit from this new approach, involving a wide spectrum of diseases, from prenatal screening to the adult CHD population [8,9,10,11]. AI mimics the ability of human intelligence to learn, analyse and make decisions. Although its first use was described in the middle of the 20th century, in the last decade, thanks to the improvement of computational technologies and their availability on a large scale, it has become widespread. It learns through algorithms, and it can be trained on specific tasks to handle complex issues in a human-like fashion [12]. The first subfield of AI is machine learning (ML), which is the ability of a system to autonomously acquire knowledge by extracting patterns from large amounts of data, defined as “big data”. 

The latter is defined by four features, which collectively highlight the role of managing and extracting values from large datasets. 

Big data are characterised by a massive amount of data generated continuously. Velocity describes the speed at which data are created and processed, often in real time. Variety describes the different types of data. Veracity highlights the importance of data quality and reliability, ensuring trustworthy insights [13]. These data are processed through a given AI algorithm, and complex analysis is performed in a few seconds, optimizing task performance [14]. 

ML algorithms, such as random forests, support vector machines and neural networks, have demonstrated efficacy in predicting various outcomes associated with CHD. They may include mortality, post-surgery complications, long-term prognosis and response to treatment. For instance, a study by P.G. Canadilla et al., employed ML techniques to predict the risk of adverse events in paediatric patients undergoing cardiac surgery, achieving high accuracy and specificity [15]. Thus, by incorporating clinical data, imaging findings and genetic markers, ML models can enhance risk stratification and aid in early intervention strategies.

## 2. Role of Statistical Tools in the Management of Congenital Heart Disease

Nowadays, predicting outcomes, stratifying the risk and personalizing the treatment and follow-up in patients with CHD involves the application of various ML models and AI tools, each with its strengths and limitations. These techniques are based on a sort of experience acquired from the analysis of large groups of actual cases, processed with different methods and expressed in a set of rules [16]. Through these new strategies derived from statistics and computer sciences, big data can be construed to find new predictive models.

Table 1 depicts some of the most important tools on which artificial intelligence, machine learning and deep learning are based.

The reported statistical tools were served through some dedicated software created to perform even very complicated statistical calculations on large amounts of data. There are usually some functions already prompting the simplest statistical tools, while for the most complex analyses, it is necessary to use some codes for each software.

The use of these statistical tools in the medical field and congenital heart disease must always be guided by clinical sense to avoid obtaining distorted results that cannot be transposed into everyday clinical reality. The main risk is represented by overfitting and collinearity [16,17]:

1. Overfitting occurs when a model captures random fluctuations or noise in the data rather than underlying patterns. In the context of CHD analysis, overfitting can lead to overly complex models that perform well on the training data but fail when generalised to real life. This can result in misleading conclusions about the relationships between variables and the prediction of outcomes. Overfitting could be mitigated by strategies like cross-validation, regularisation and feature selection to ensure that the model is not overly sensitive to noise in the data and could be generalised well to new patients.
2. Collinearity turns up when two or more predictors are highly correlated with each other in a regression model. In CHD analysis, collinearity can distort the estimated coefficients, making it difficult to assess their effect on the outcome. Collinearity can arise due to the complex interplay of genetic, environmental and clinical factors influencing the development and progression of the disease. To address collinearity, researchers can perform diagnostic tests such as variance inflation factor analysis to identify highly correlated predictors. Moreover, methods such as variable transformation or variable selection could be used to mitigate the effects of collinearity.

The critical points of the described statistical methods can also vary depending on the specific dataset, the complexity of the problem and the implementation details. Additionally, practical considerations such as data quality, feature engineering and model tuning can significantly impact the performance of algorithms.

## 3. Application of Artificial Intelligence in Diagnosis, Management and Follow-Up of CHD

### Cardiac Imaging Evolution: Artificial Intelligence-Guided Advancement

Echocardiography, computed tomography (CT) and cardiac magnetic resonance (CMR) imaging are, respectively, the first- and second-level imaging tools in paediatric cardiology. 

The revolution in medical imaging started about 30 years ago, from image printing to the digitalisation of exams. Firstly, the computer was used to modify images in terms of gain, contrast and cine loops. In the past two decades, software has started to offer algorithms for quantification and/or automatic report generation. 

Recently, AI-derived technologies have been increasingly used to analyse standard cardiac imaging data, aiding in the diagnosis and monitoring of CHD [29,30,31]. DL models can automatically detect and classify abnormalities in cardiac images, assisting clinicians in assessing disease severity and planning interventions [32,33]. 

Oktay et al. presented an approach for the precise localisation of anatomical landmarks in cardiac images using stratified decision forests. The authors addressed the challenge of accurately identifying key landmarks in cardiac images, which serve as reference points for subsequent image analysis tasks and are critical for accurate interpretation and diagnosis [34]. 

**Figure 1:**
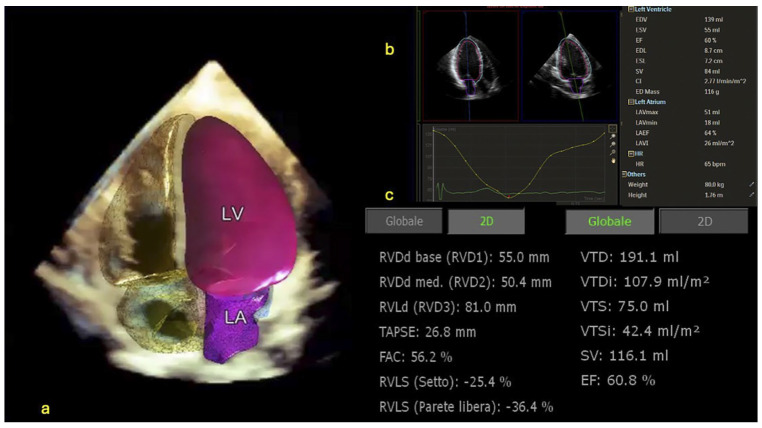
3D evaluation by Dynamic Heart Model (Philips). (**a**) The software automatically calculates left ventricle (LV), right ventricle (RV), left atrium (LA) and right atrium (RA) volumes and functions; (**b**) LV and LA reports, including graphs of LA and LV volume changes during the cardiac cycle; (**c**) Data report of the right ventricle. The system generated volumes and ejection fractions. In addition, it automatically calculates fractional area change (FAC), tricuspid annulus peak systolic excursion (TAPSE) and RV strain values, usually calculated by 2D images. Thus, by using a single image, a complete report on the RV function can be generated.

The goal of AI is to work in a human-like fashion. In this regard, Tison et al. presented an ML algorithm capable of automated echocardiographic quantification of left ventricular ejection fraction without identifying endocardial borders and volume measurements, mimicking the expertise of human readers [35], Figure 1. AI tools can detect subtle abnormalities in valve structure and function, allowing early detection of valvular disease [36].

In addition, ML has also been used to assess valvular and anatomic pathology, as well as to enhance the quality of existing echocardiograms [37]. 

In the field of foetal cardiology, Veronese et al. documented that 3D printed models are a practical instrument for healthcare providers to enhance parental understanding of a child’s cardiac anomaly and facilitate the formulation of subsequent surgical strategies [38].

**Figure 2:**
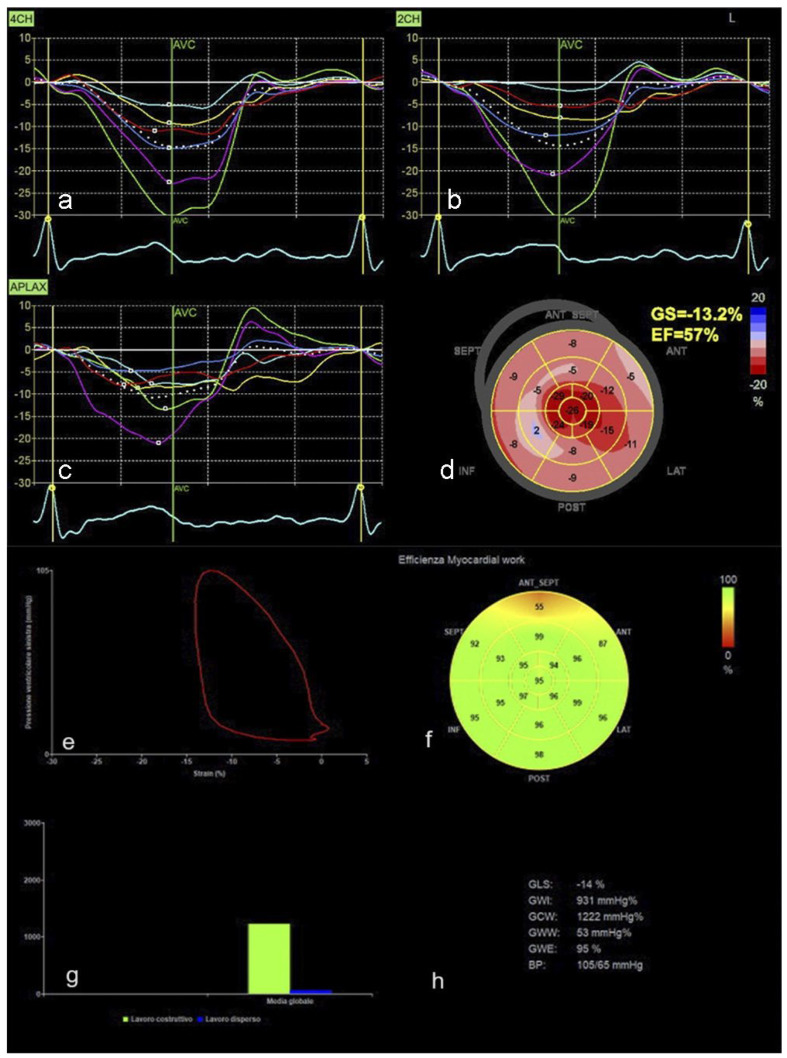
Global longitudinal strain (GLS) and GLS-based myocardial work (MW) software (General Electrics, Boston, MA, USA) by 2D echocardiography in a heart transplanted patient. Results were reported in tables, graphs or plots. GLS is calculated by three apical views, generating 6 plots for each view (**a**–**c**); GLS can be also visualised in a bull’s eye plot, displaying 17 segments (**d**); Left ventricle (LV) myocardial work is based on pressure–strain loop (**e**); the software can calculate global MW and segmental MW, in the latter case, it can be visualised in a 17 segments bull’s eye (**f**); segmental analysis allows to study MW efficacy: constructive (green bar) and wasted (blue bar) global work. These data can be displayed in graphs (**g**); or tables (**h**).

In addition, by automating image segmentation, feature extraction and 3D reconstruction, AI facilitates personalised treatment strategies tailored to each patient’s unique cardiac anatomy [29], as shown in Figure 2. Segmentation refers to recognising a structure in the framed image, marking its contours and performing measurements. It can be employed in the assessment of ventricular size to quantify chamber volumes and determine ejection fraction [39]. In addition, it can be performed in the valvular annulus, leaflets/cusps and even Doppler and speckle tracking analysis [40].

Davies et al. provided an insightful examination of DL techniques in the field of CHD imaging [41].

The authors highlighted how algorithms and computational sciences might change cardiac imaging, allowing a less time-consuming imaging analysis (e.g., by measuring structures or plotting cardiac boundaries) towards a more reliable image interpretation.

## 4. Artificial Intelligence in Clinical Decision-Making: From Datasets to Solutions 

The concept of computer-based clinical decision-making dates back to the 1960s. The progressive digitalisation and spread of communication technologies encouraged the creation of software and predictive models that integrate patient data to facilitate clinical decisions [42]. In this context, clinical decision support systems (CDSS) emerged as a valuable tool to assist paediatric cardiologists in providing optimal care. 

These systems provide recommendations for diagnostic testing, treatment options and follow-up plans tailored to the patient’s CHD subtype, comorbidities and risk factors.

The application of CDSS in everyday practice enhances accuracy because the design and framework of the software assist clinicians in making more accurate and evidence-based decisions, reducing the risk of diagnostic errors and inappropriate treatments.

Furthermore, by automating routine tasks such as data analysis and guideline adherence checks, CDSS saves time for clinicians, allowing them to focus more on patient care and complex decision-making.

Shah et al. [43] provide valuable insights into the challenges and considerations associated with making ML models clinically useful. By emphasising the importance of clinical relevance, interpretability, workflow integration, validation, regulatory compliance and stakeholder engagement, the authors offer a framework for effectively translating ML research into actionable insights that improve patient care in real-world healthcare settings. 

## 5. Artificial Intelligence and Outcome Prediction

One of the most important applications of AI is predicting outcomes and personalising follow-up for patients with CHD through the development of predictive analytics and decision support systems [44]. 

AI techniques, such as ML and DL, are applied to large datasets containing clinical, imaging and genomic data of CHD patients. These models predict various outcomes, such as mortality, hospital readmission, disease progression and response to treatments [45].

H. Moroz et al. developed an attention-based deep-learning model, hART (heart failure Attentive Risk Trajectory), which accurately identifies high-risk individuals and predicts heart failure (HF) risk trajectories over time [46]. The model incorporates diverse clinical variables, demographic factors and temporal information to generate personalised risk assessments. The ability to forecast individualised risk plots makes it a promising tool in the management of HF risk, moving from general Kaplan–Meyer plots to individualised plots. Thus, counselling as well as follow-up might be personalised and major event risks can be adequately predicted. Paediatric cardiology is a polyhedric speciality: once approaching a child affected by CHD, issues about individual variability in anatomical and physiological characteristics should be considered. Tetralogy of Fallot (TOF) could be a paradigmatic example: it moves from “pink Fallot” to pulmonary atresia and pulmonary vessel anomalies. Thus, its heterogeneous nature raises challenges in predicting the kind of surgery and long-term outcomes [47]. In post-operative settings, factors such as residual pulmonary stenosis, right ventricular dysfunction and arrhythmias all contribute to the different clinical courses observed in TOF patients. Multi-modality imaging is largely available in these patients; however, the appropriateness and the merging of information obtained from different imaging modalities might be not easy for physicians, especially for beginners or for low-volume centres. In these settings, DL imaging analysis might offer a powerful approach to enhance prognostic prediction in TOF patients. By rapidly analysing cardiac images, AI algorithms assist in precise diagnosis and characterisation of TOF, including identifying key anatomical features such as ventricular septal defects and overriding aorta, thus enhancing optimised surgical planning.

Diller et al. demonstrated that DL models can provide valuable insights into disease progression, guide treatment decisions and improve long-term outcomes by leveraging the wealth of information contained within cardiac imaging data [48].

Aortic Coarctation (AoC) diagnosis is still a challenge in foetal cardiology and perinatal settings. Despite advancements in medical imaging and diagnostic techniques, the diagnosis of AoC often requires careful interpretation of imaging and skilled operators. Despite all this, also in evolving National Healthcare Systems, undiagnosed AoC still remains a reason for infant death [49]. AI-based tools offer a transformative approach to aid in the diagnosis of AoC by leveraging ML algorithms to analyse complex datasets and identify subtle patterns indicative of the condition [50]. These algorithms can detect variations in aortic diameter, blood flow velocities and wall thickness, aiding in the early and precise diagnosis of the condition.

The utility of novel DL algorithms in complex CHD was first demonstrated by Diller et al. [51]. The paper emphasises how ML tools trained on routine echocardiographic datasets might revolutionise the way to manage patients affected by any complex congenital cohort. 

By harnessing the power of data-driven analytics, ML algorithms enable automated segmentation of ventricular areas and offer a holistic approach to risk assessment, ensuring adaptability to evolving patient characteristics and treatment paradigms.

Naruka et al. systematically reviewed the role of AI and ML in predicting graft failure and mortality after heart transplantation (HT) [52]. In this paper, the authors depicted how AI facilitates the integration of multi-omics data, including genomics, transcriptomics, proteomics and metabolomics, to gain a comprehensive understanding of the molecular mechanisms underlying cardiac graft failure.

By investigating mortality rates and the most predictive variables of transplant success, the authors underlined the superiority of novel tools compared to traditional regression models and multivariable analysis [53]. In addition, AI offers valuable insights into prognosis and aids in optimising transplant protocols [54]. The United Network for Organ Sharing (UNOS) database was analysed using AI to find ML-derived risk models for the prediction of mortality in adults with CHD undergoing HT [55]. Delving into the depths of the UNOS database, AI-driven algorithms dissect a vast amount of patient information and transcend mere data analysis, unravelling intricate patterns and correlations that elude and overcome traditional analytical methods.

In this regard, by automatically quantifying histological features such as inflammation, fibrosis and endothelial damage, AI algorithms can expedite the interpretation of biopsy slides and provide objective assessments of graft health.

By using a large multicentre clinical dataset, Smith et al. demonstrated the potential of ML models to accurately predict five-year transplant-free survival rates among infants with hypoplastic left heart syndrome, thus allowing risk stratification of affected patients [56]. Moreover, the authors highlighted how AI-driven image analysis enhances reproducibility and standardisation across different pathology laboratories, ensuring consistency in graft surveillance and management.

This comprehensive assessment encompasses demographic factors, clinical parameters and previous surgical history, thus enabling the creation of ML-derived risk models endowed with unparalleled predictive accuracy. 

## 6. Artificial Intelligence to Unveil Omics Medicine 

The impact of genetics on CHD is of straightforward importance, mostly for those affected by extra-cardiac anomalies. Advances in biosciences have allowed a better understanding of human genes, with a large amount of data that needs to be interpreted. AI techniques excel at analysing large volumes of complex data, like genomic and transcriptomic data, to identify genetic variants associated with CHD susceptibility, severity and treatment response [57]. These algorithms can efficiently analyse vast amounts of genetic data, identifying genetic variations that are likely to have functional significance and contribute to the development of CHD. By analysing multi-omics data, including genomics, transcriptomics, metabolomics and proteomics, AI can uncover molecular signatures indicative of disease states or treatment responses [58]. In particular, AI allowed the birth of these branches, because it is able to integrate thousands of genetic and “omics” information with clinical variables [59]. The aim of these branches is to create a genetic and/or metabolic/proteomic fingerprint of the patients. By comparing patients’ data, it is possible to identify specific pathways that may become therapeutic targets for personalised care. Similarly, these new sciences might predict the development of diseases since foetal age, or, in borderline cases, discriminate the outcome of these patients [60,61]. 

Feng et al. employed facial recognition software by using AI to recognise patients affected by cardio-facial-cutaneous syndromes [62]. Employment of AI tools (Face2Gene) and the development of automated image analysis systems (Face2Gene RESEARCH) have been shown to help with facial recognition and genotype identification in patients affected with Noonan syndrome, thus improving diagnostic accuracy [63]. 

Ameen et al. displayed single-cell RNA sequencing (scRNA-seq) to analyse gene expression profiles at different stages of cardiogenesis by using ML techniques to interpret the vast amount of generated data [64]. Novel tools are useful in analysing complex biological data to gain insights into developmental processes and ultimately to understand cardiogenesis.

Lin Ling et al., explored the molecular signatures and systematic changes in heart function of end-stage dilated cardiomyopathy (DCM) using anatomically resolved transcriptome and proteome landscapes [65]. The most relevant findings include insights into protein expression patterns contributing to DCM and their correlation with functional alterations in the heart. In addition, a comprehensive transcriptomic profile was used to reveal gene expression alterations with potential implications for prognosis and therapeutic targeting in DCM.

## 7. Insights of Artificial Intelligence in Continuous Monitoring

The landscape of healthcare has been significantly transformed by telemedicine. Among these innovations, remote monitoring and wearable devices have emerged as powerful tools with the potential to enhance the care and outcome of individuals with CHD outside hospital settings [66,67,68].

ML algorithms analyse data to detect signs of deterioration, arrhythmias, or other cardiovascular events, triggering timely interventions and preventing hospitalisations.

Telemedicine might reduce in-hospital stays, prevent clinical deterioration and inter-admission mortality and at the same time allow therapy optimisation. This tool might be particularly helpful for patients living far from referring hospitals or in low-income countries where it is difficult to promptly offer specialised support. Integration of telemedicine platforms and remote monitoring devices in the standard clinical practice of CHD augments follow-up care and patient engagement while mitigating healthcare inequalities.

## 8. Artificial Intelligence and Machine Learning Applied to Congenital Cardiac Surgery and Interventional Cardiology

The integration of AI tools with imaging technology allows better visualisation of complex congenital anatomy and improves the assessment of its physiology. These aspects are increasingly important in the complexity of congenital heart surgery and catheter-based procedures [69]. 

Before surgical procedures, the operators might simulate the operation using AI. This tool can be used to plan surgery and to verify alternative approaches before “real surgery” [33]. In addition, it can be used for teaching purposes for students and fellows. AI-powered surgical simulators replicate realistic surgical scenarios, allowing surgeons to practice procedures in a risk-free virtual environment. ML algorithms can enhance simulation realism by providing dynamic tissue deformation, patient-specific anatomy and real-time feedback on surgical performance [70]. This is particularly helpful in low-volume centres, because it allows to repeat the manoeuvre several times, improving and getting faster the learning curve of fellows and operators. 

Three-dimensional (3D) printing can help during the planning of a catheter-based intervention by transforming a digital render into a physical tangible form of complex anatomy [71]. Several studies demonstrated that 3D printing improved the comprehension of CHD in students and improved counselling on complex CHD in foetal cardiology [32,38].

Virtual reality (VR) and augmented reality (AR) are new visualisation options in the cardiovascular field.

VR immerses surgeons in realistic surgical scenarios using interactive virtual 3D environments [72,73,74]. Conversely, the AR system overlays virtual information in a real-world environment by transposing anatomical structures, surgical plans and intraoperative guidance into the surgeon’s field of view [42,75]. Cattapan et al. performed an observational study involving cardiac surgery residents to assay the role of surgical simulation on 3D cardiac models as a promising innovative tool to improve understanding of cardiac anatomy, facilitate hands-on learning and enhance proficiency in surgical techniques and confidence levels when performing cardiac surgery [76]. Towards this trajectory, AI-driven continuous learning systems encourage ongoing skill development and provide personalised feedback and insights for surgeons [69].

During surgical procedures, AI-powered systems provide real-time guidance and decision support to cardiac surgeons and interventional cardiologists. By integrating intraoperative imaging modalities, physiological data and predictive analytics, AI algorithms assist in navigating complex anatomical structures, optimising device placement, and minimising procedural complications [77]. Furthermore, AI-driven outcome prediction models analyse preoperative patient data, surgical parameters and postoperative outcomes to identify predictive factors associated with surgical success or complications. Moreover, ML algorithms can assist surgeons in preoperative planning and risk stratification, enhancing patient selection and optimising surgical outcomes [15,29]. 

On the other hand, during interventional cardiology procedures, the continuous visualisation of anatomical structure is crucial. In this field, AI can integrate information from different types of imaging to clarify the real-time representation of anatomical structures [78]. 

Dealing with CHD from a surgical point of view is quite challenging because of the high surgical expertise required, the long learning curve and safety concerns. One of the most important applications of AI and ML to improve the learning curve of congenital heart surgeons is through the development of surgical simulation and decision support systems [78]. 

## 9. Exploring Boundaries: Challenges in Applying Artificial Intelligence

Nowadays, the application of novel tools with powerful algorithms and computing capabilities holds significant promise in the diagnosis, treatment, and management of CHD (Table 2).

Despite the transformative potential of AI and ML several challenges and ethical considerations warrant attention [77]. These issues concern data privacy and security, algorithm bias and fairness, transparency and interpretability, clinical integration and workflow adaptation [51]. In addition, regulatory human oversight is mandatory to avoid the complete delegation of decision-making authority to AI. 

The European Union is the first country worldwide to take significant steps towards establishing regulations for AI systems to ensure they are developed and deployed in a trustworthy manner. The AI Act was approved on 13 March 2024 and aims to address various concerns related to AI, including safety, fairness and accountability [79].

The AI Act proposal underlines that digital evolution must be shaped with full respect for fundamental rights so that digital technologies might serve humanity.

Addressing these issues requires collaborative efforts among clinicians, data scientists and regulatory bodies to ensure the development of robust and clinically relevant prognostic models.

Some innovative methods are in their early stages of experimentation and require additional refinement before achieving widespread acceptance in routine clinical applications [69]. For example, echocardiographers often select single frames for manual segmentation to avoid foreshortening of cardiac chambers or variation from an irregular heart rhythm, which could affect the analysis. Addressing such tasks for an automated system is challenging. 

As an example, low frame rate implies reduced temporal resolution and motion artefacts leading to poor image quality and thus hindering the performance of AI-based algorithms. 

In scientific research, the use of AI involves issues about intellectual honesty and the trustworthiness of papers. M. Majovsky et al. highlighted how AI algorithms and generative models can generate high-quality fraudulent works [80]. These models can unintentionally facilitate plagiarism and cheating by generating contents that closely resemble existing works without paper attribution, resulting in fraudulent copyright that is not the result of human thinking [81]. Furthermore, generative AI tools can be trained on sensitive data, which can lead to the unauthorised use or disclosure of sensitive information. 

## 10. Conclusions and Future Directions

The integration of AI and ML into the management of CHD involves a transformative shift. Current AI algorithms analyse large volumes of data, make pretty reasonable interpretations of results and could even suggest possible diagnoses and therapeutic decisions, thus saving time, material, financial and human resources. CHD, although not rare, is not among the everyday-encountered cardiovascular affections. On the other hand, it is a very large and heterogeneous group of diseases, and gathering a sufficient level of clinical expertise may be challenging for many clinicians. 

The application of artificial tools could significantly facilitate the diagnostic and decision-making process by improving risk stratification and longitudinal follow-up.

Leveraging AI advancements can refine CHD diagnosis, ensuring higher precision, efficacy and accessibility. In addition, patients may receive personalised care, overcoming the boundaries of conventional medicine.

Implementation of education and training initiatives is straightforward for widespread adoption and effective employment of new technologies in CHD management.

AI raised concerns about privacy, informed consent, scientific conceptualization and intellectual properties. Technologies usually go faster than national and international laws and regulations. 

In the very near future, AI might be claimed to fight AI itself. 

## Figures and Tables

**Table 1:** Artificial intelligence and machine learning tools.

Tools	Applications in Congenital Heart Disease	Advantages	Disadvantages	References
**Logistic Regression** Analysis of variables related to binary outcomes	Prediction of mortality or hospital readmission based on clinical data, or reintervention in patients treated by surgery	Suitable for binary classification tasks Easily interpretable results	Performance degrades with many variables or collinearity between them Accurate selection of variables is required to minimise the risk of overfitting	[16,17]
**Decision trees** Series of hierarchical decisions (nodes of the tree) based on features	Prediction of outcomes of congenital heart disease or after surgery Identification of predictors	Easily to understand and interpret Capture non-linear relationships Handling missing value	Risk of overfitting Instability to small changes in the data Bias towards features with a lot of levels	[18,19]
**Random Forests** Multiple decision trees for classification or prediction	Outcome prediction based on clinical data	Automated feature selection Less prone to overfitting compared to individual decision trees Elaboration of high amounts of data	May require substantial computational resources Black-box nature makes interpretation challenging	[16,20,21]
**Gradient Boosting Machines** Sequentially decision trees	Comprehension of complex feature interactions	Effective for capturing complex interactions Sequential model building corrects errors of previous models	Maybe computationally intensive Requires careful parameter tuning	[22]
**Survival Analysis Models** Cox proportional Hazard models or Kaplan-Meier estimators	Survival rates analysis Time-to-disease progression analysis	Suitable for time-to-event data analysis Non-parametric methods Can incorporate censored data	May require large sample sizes for accuracy	[21,23]
**Neural Networks** Interconnected nodes inspired by the structure of the human brain	Improvement of accuracy prediction of outcomes Deep learning in imaging analysis and simulation	Automated learning of intricate patterns and relationships Suitable for large datasets State-of-the-art performance in various domains	Requires substantial computational resources May suffer from overfitting Interpretability can be challenging	[24]
**Support Vector Machines** Algorithms for dichotomous classification	Outcome prediction	Suitable for both linear and non-linear classification tasks Provides robustness against overfitting Efficient for high-dimensional data	Training time may increase significantly with the number of variables Limited to dichotomous classification	[25]
**Long Short-Term Memory (LSTM)** Algorithms with recurrent nodes, memory cells and gating mechanisms	Analysis of time-series measurements for future outcome prediction	Suitable for sequential data analysis Minimises time lags Solves long time-lag tasks	Requires labelled data for training May require substantial computational resources Interpretability can be challenging	[26]
**Bayesian Networks** Models of probabilistic dependencies between variables	Personalised risk assessment and decision support	Represents variable dependencies using a directed acyclic graph Incorporates prior knowledge into risk assessment	May require expert knowledge for model construction Complex algorithms may be challenging to implement	[27,28]

**Table 2:** Major findings and the type of employed artificial intelligence tool in the most relevant cited studies.

Study	Year of Publication	Key Findings	AI Algorithm/Model Used
Narula et al. [37]	2016	- Automated morphological assessments (chamber size and wall thickness measurements) - Functional assessments (ejection fraction calculation)	Support vector machine Random forests Artificial neural networks
Oktay et al. [34]	2017	Anatomical landmark localisation in cardiac imaging	Stratified decision forests
D. Medvedofsky et al. [39]	2018	Three-dimensional echocardiographic quantification of left-heart chambers	Automated adaptive analytics algorithm
X. Li et al. [63]	2019	Analysis of the molecular and phenotypic spectrum of Noonan syndrome in Chinese patients	Neural networks
F. M. Asch et al. [35]	2019	Automated quantification of left ventricular ejection fraction without volume measurements	Neural network
G.P. Diller et al. [51]	2019	- Outcome prediction in the systemic right ventricle (sRV); - Risk stratification in sRV - Management in sRV	Convolutional neural networks
Y. Lu et al. [78]	2020	Perform CT-TEE image registration for surgical navigation of congenital heart disease	Cycle adversarial network (deep learning)
F. P. Lo Muzio et al. [77]	2021	Support decision-making in cardiac surgical practice	Supervised machine learning
M. Mann et al. [59]	2021	Facilitate proteomics and biomarker discovery to improve disease diagnosis and therapeutic targeting	Artificial neural networks
B. Ayers et al. [54]	2021	Development of predictive models to improve survival prediction after heart transplant	Artificial neural network, Support vector machine Random forest
Y. Li et al. [60]	2021	Identification of biomarkers for CHD using maternal amniotic fluid metabolomics	Logistic regression
Nedadur et al. [36]	2022	- Accurate classification and grading of valvular heart disease severity; - Automated detection of valve morphology abnormalities	Convolutional neural network (CNN)
V. Naruka et al. [52]	2022	Identification of AI-driven approaches for risk prediction, patient selection and post-transplant outcomes assessment	Deep neural networks
M. Michel et al. [58]	2022	- Identification of metabolic biomarkers associated with CHD - Development of a predictive model for patient stratification	Random forests
B. Feng et al. [62]	2023	Analysis of the molecular and phenotypic spectrum of cardio-facio-cutaneous syndrome in Chinese patients	Neural networks
Li Lin et al. [65]	2023	Identification of disease-relevant molecular signatures and changes in heart function in dilated cardiomyopathy	Neural networks
M. Ebrahimkhani et al. [68]	2023	Analysis of wearable seismocardiography (SCG) data for diagnosing aortic valve stenosis and predicting aortic hemodynamics obtained by 4D flow MRI.	Convolutional neural network
P. N. Kampaktsis et al. [55]	2023	Development of a risk-prediction model for assessment of 1-year mortality post-heart transplantation	Logistic regression Adaptative boosting Random forests
H. Morotz et al. [46]	2024	Prediction of lifespan heart failure risk trajectories	Logistic regression Support vector machine
